# Dermatitis caused by Ctenocephalides felis (cat flea) in human

**Published:** 2014

**Authors:** Mohammad Reza Youssefi, Soheil Ebrahimpour, Mojtaba Rezaei, Ehsan Ahmadpour, Arash Rakhshanpour, Mohammad Taghi Rahimi

**Affiliations:** 1 Department of Parasitology, Islamic Azad University, Babol Branch, Babol, Iran.; 2 Infectious Diseases and Tropical Medicine Research Center, Babol University of Medical Sciences, Babol ,Iran.; 3 Young Researchers Club, Islamic Azad University, Babol Branch, Iran.; 4 Department of Medical Parasitology and Mycology, School of Medicine, Mazandaran University of Medical Sciences, Sari, Iran.; 5 Department of Medical Parasitology and Mycology, School of Public Health, Tehran University of Medical Sciences, Tehran, Iran.

**Keywords:** Ctenocephalides felis, Flea, Dermatitis, Skin, Human, Iran

## Abstract

***Background:*** Human infestation to ectoparasites such as ticks, lice, cimex, fleas, mites and others agents may result in intensive allergic reaction with symptoms of itching, skin infection and severe irritation. In this case report, we present a case of dermatitis caused by cat flea.

***Case presentation:*** A three-member family referred to dermatology clinic in Babol due to dermal complications. They complained of irritation and the unrest caused by intense itching. Samples of tiny live insects were detected from their clothing which was recognized as C. felis (cat flea).

***Conclusion: ***This report highlights the importance of ectoparasites causing dermatitis.

Ectoparasitic disease including scabies, pediculosis, demodicosis and others. are caused by arthropods which inhabit primarily on the surface of the host. One of the major hostiles insect pests are fleas that transmit a variety of viral, bacterial and rickettsial diseases to humans and animals (1). When fleas pierce the skin of the host with their highly specialized mouthparts, a phenomenon called Flea Allergy Dermatitis (F.A.D.) occurs originating from substances in flea saliva due to flea bite (2). Scratching and itching are the most agonizing symptoms of flea bites in host. Flea bites cause itchy papules, which are often in groups of three, the so-called breakfast, lunch and dinner configuration (3). Here, we point to reports attributed to human infestation by fleas in the different parts of the globe: In Switzerland, a couple was attacked by pigeon fleas (*Ceratophyllus columbae*) and the patients showed allergic urticarial reaction to bites (4). In another similar report, a young woman was invaded repeatedly by cat flea from suburban raccoons (5). In some cases, fleas had been found by staff in their uniforms and all family members had been bitten at home (6). The most likely species to bite humans are C. felis felis or (Xenopsylla cheopis). Despite this fact, C. f. felis is an obligate ectoparasite whose main hosts are cats (7). The presence of fleas in surrounding areas threats human life and increases the probability of disease transmitted by them (8). Therefore, the current report describes human infestation by ectoparasites causing cutaneous severe reactions and dermatitis. 

## Case presentation

A family of three members, a man with his spouse and a son who live in Gorgan City, Golestan province, Iran referred to the dermatology clinic in Babol complaining of multiple bites accompanied by severe itching in the different parts of their body especially, the legs. Some samples of tiny live insects were collected from their clothing and the infected clothes were taken for examination. 

**Figure 1:**
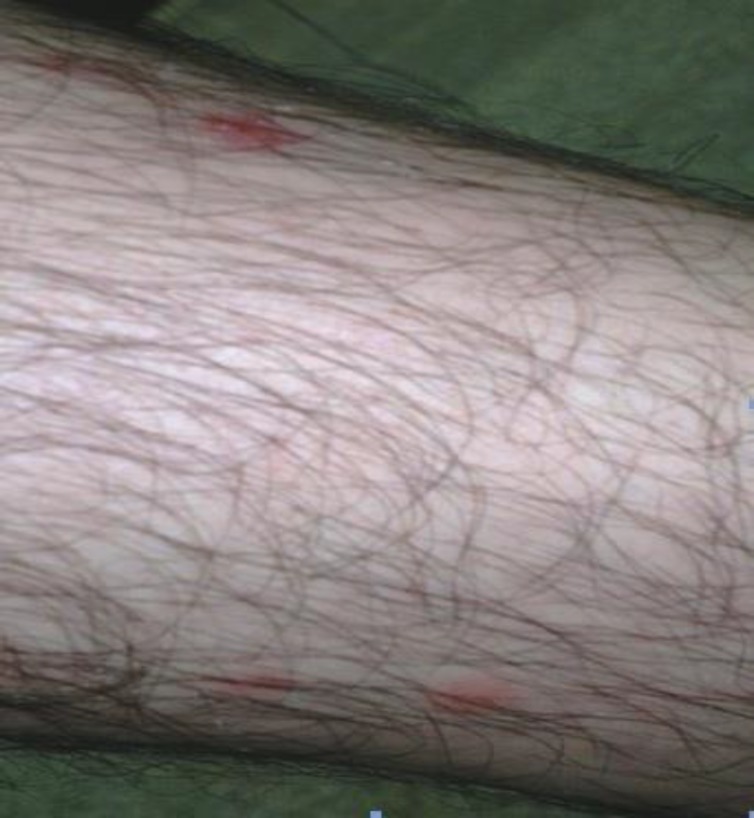
Multiple lesions and solar erythema due to flea bites in the leg of a 32 year old man

They complained of irritation and intense itching. The sites of scratching were tender and painful, particularly at night. Examination of the affected skin demonstrated many papules with solar erythema measuring 2-10 mm in diameter, the affected areas were vivid in red color by which they were surrounded with bright pink areola about 2 mm wide. Furthermore, in the center of some of the bites, a red hemorrhagic punctum was observed. Most lesions were in groups of three (figure 1).

Further investigation revealed that their neighbors enjoy keeping pets and domestic animals and also recently they have been on close contact with them. After careful examination and scrutiny of the collected minute samples, the samples were sent to the Department of Parasitology of the lslamic Azad University where the diagnosis of c.felis was made (cat flea). Treatment began by applying hydrocortisone cream as an antipruritic twice a day and all the patients’ symptoms fade after 12 hours and disappeared after four days. The elimination of fleas from the house yard, furniture and all suspected locations was accomplished by spraying and fumigating with simetrin. 

## Discussion

In this case report, we present a case of dermatitis caused by cat flea. Cat flea saliva, feces and debris are considered as allergens (9). Usually in late summer (August and September) flea populations reach their peak and their potential ability to attack to host increases when the temperature and humidity increases which is compatible to our ealier report (10). Concerning the delayed reaction in both males and females, younger age groups were noticeably more sensitive compared to older age groups to the bites of fleas in one study (11). Similar to our case, other researchers also reported dermatitis due to bites flea (4-6).

Based on the characteristic patterns of flea bites, they normally prefer the ankles and bare feet while in the current report, most of bites were limited to the patient’s foot (12). The skin reaction to insect bites and stings usually last only up to few days. Nonetheless, in some cases, the local reaction of skin can last up to two years. These bites sometimes cause misdiagnosis in other forms, as either benign or cancerous lesions (13). Not only flea bites may result in severe allergic reactions in susceptible individuals but also scratching the bites can lead to secondary infection. It is possible that the prevalence of human infestation due to arthopods in North of Iran is high and the patients were misdiagnosed as nonspecific dermatitis. Hence, more studies on ectoparasites and human infestation in the north of our country due to its appropriate climate and geographical conditions are highly recommended.

In conclusion, this case presentation in our region highlights the importance of ectoparasites causing dermatitis. 
